# Biogeographical and seasonal dynamics of the marine Roseobacter community and ecological links to DMSP-producing phytoplankton

**DOI:** 10.1038/s43705-022-00099-3

**Published:** 2022-02-14

**Authors:** James O’Brien, Erin L. McParland, Anna R. Bramucci, Nachshon Siboni, Martin Ostrowski, Tim Kahlke, Naomi M. Levine, Mark V. Brown, Jodie van de Kamp, Levente Bodrossy, Lauren F. Messer, Katherina Petrou, Justin R. Seymour

**Affiliations:** 1 Climate Change Cluster, University of Technology Sydney, Broadway, NSW Australia; 2 School of Life Sciences, University of Technology Sydney, Broadway, NSW Australia; 3 Department of Marine Chemistry and Geochemistry, Woods Hole Oceanographic Institution, Woods Hole, MA USA; 4 Department of Biological Sciences, University of Southern California, Los Angeles, CA USA; 5 School of Environmental and Life Sciences, University of Newcastle, Callaghan, NSW Australia; 6 CSIRO Oceans and Atmosphere, Hobart, TAS Australia; 7 Centre for Microbiome Research, School of Biomedical Sciences, Queensland University of Technology, Brisbane, QLD Australia

**Keywords:** Microbial ecology, Microbial ecology

## Abstract

Ecological interactions between marine bacteria and phytoplankton play a pivotal role in governing the ocean’s major biogeochemical cycles. Among these, members of the marine Roseobacter Group (MRG) can establish mutualistic relationships with phytoplankton that are, in part, maintained by exchanges of the organosulfur compound, dimethylsulfoniopropionate (DMSP). Yet most of what is known about these interactions has been derived from culture-based laboratory studies. To investigate temporal and spatial co-occurrence patterns between members of the MRG and DMSP-producing phytoplankton we analysed 16S and 18S rRNA gene amplicon sequence variants (ASVs) derived from 5 years of monthly samples from seven environmentally distinct Australian oceanographic time-series. The MRG and DMSP-producer communities often displayed contemporaneous seasonality, which was greater in subtropical and temperate environments compared to tropical environments. The relative abundance of both groups varied latitudinally, displaying a poleward increase, peaking (MRG at 33% of total bacteria, DMSP producers at 42% of eukaryotic phototrophs) during recurrent spring-summer phytoplankton blooms in the most temperate site (Maria Island, Tasmania). Network analysis identified 20,140 significant positive correlations between MRG ASVs and DMSP producers and revealed that MRGs exhibit significantly stronger correlations to high DMSP producers relative to other DMSP-degrading bacteria (*Pelagibacter*, SAR86 and *Actinobacteria*). By utilising the power of a continental network of oceanographic time-series, this study provides in situ confirmation of interactions found in laboratory studies and demonstrates that the ecological dynamics of an important group of marine bacteria are shaped by the production of an abundant and biogeochemically significant organosulfur compound.

## Introduction

Roseobacters are a globally ubiquitous group of heterotrophic bacteria found in marine surface waters [1]. In the open ocean, the marine Roseobacter Group (MRG) generally represents <8% of bacterial cells [2, 3], but in more productive coastal waters they are often dominant members of bacterial communities, particularly during phytoplankton blooms [4–12], and can comprise up to 25% of all bacteria [13, 14]. Roseobacters have been described as archetypical phytoplankton associates [15] and have been shown to be key players in the transformation of phytoplankton-derived dissolved organic matter (DOM), as well as potential mutualistic partners of some phytoplankton species [7, 16].

The ecological interactions between members of the MRG and phytoplankton have been shown to be underpinned by sometimes reciprocal exchanges of a diverse suite of organic molecules [7, 16]. Among these, organic sulfur compounds, including the phytoplankton secondary metabolite dimethylsulfoniopropionate (DMSP), are believed to be key currencies within MRG–phytoplankton interactions [17, 18]. DMSP is produced in large quantities by several marine phytoplankton species, with this single compound accounting for up to 10% of photosynthetically-derived carbon in the surface ocean [19, 20]. DMSP is consequently an important source of DOM for bacterial heterotrophy in the surface ocean, where it has been estimated to contribute up to 13% of carbon and 100% sulfur requirements of some marine bacteria [21, 22]. The MRG, like other abundant lineages of marine bacteria, including *Pelagibacter* (HTCC1062), can metabolise DMSP using multiple pathways. There are two primary degradation pathways for DMSP [23]: the DMSP lyase pathway, which cleaves DMSP to produce acrylate and the volatile gas, dimethylsulphide (DMS); and the DMSP demethylation pathway, which precludes DMS as a biproduct and results in the assimilation of DMSP-derived sulfur. The balance between these two pathways, which is largely determined by the composition and behaviour of the bacterial community, has profound biogeochemical implications [24]. This is because the ventilation of DMS (derived from the DMSP lyase pathway) from marine surface waters represents the largest source of biogenic sulfur in the atmosphere, where it can ultimately be converted to cloud condensation nuclei that increase albedo [25]. The distribution of the two DMSP degradation pathways among marine bacteria is not equal, as DMSP demethylation is more widespread than lyase [26], but the relative proportion of these genes can shift dependent on compositional changes in the bacterial community (including increases in members of the MRG) [27]. Furthermore, even within a given Roseobacter strain, there is evidence that the relative expression of these two genes can vary substantially according to different environmental conditions [28]. Cumulatively, changes in the relative occurrence and expression of these DMSP degradation pathways will potentially be a critical factor governing oceanic DMS production.

The important role of the MRG in marine sulfur cycling has been widely documented in laboratory-based studies, whereby isolates have been shown to grow from DMSP enrichment and subsequently produce DMS and acrylate [29], and have been reported to establish mutualistic associations with DMSP-producing phytoplankton [7, 16]. Members of the MRG have been shown to contain genes encoding DMSP demethylation (*dmdA*) and multiple lyase genes (*dddD,L,P,W* and *Q*) [30–34]. Evidence for the role of DMSP in MRG–phytoplankton interactions has also been inferred from short-term field-based studies that have reported MRGs as a dominant feature of bacterial communities during blooms of DMSP-producing phytoplankton [4, 10, 11, 35, 36].

The temporal and spatial distribution of the MRG has been very well characterised as a group, but recent focus on 16S rRNA gene sequences has shown that differences in biogeographic and seasonal patterns are evident when examining species or subclades of the MRG [37–39]. For instance, the CHAB-I-5 subclade occurs globally, from tropical to polar surface waters and represents a major component of the total bacteria community in the North Sea and Atlantic Ocean in summer [14, 40]. However, there is little evidence that this cluster is interacting with marine phytoplankton, i.e. there is no significant correlation between CHAB-I-5 and chlorophyll *a* and members are almost exclusively found in free-living fractions of bacterial communities [14]. Conversely, another MRG subclade, the Roseobacter clade affiliated (RCA) cluster, are notably absent in tropical and subtropical waters, but are among the most abundant bacterioplankton in temperate to (sub)polar environments, where their abundance is often positively correlated with chlorophyll *a* [13].

Emerging differences in biogeography, temporal dynamics and life-history traits within the MRG highlight the need to improve our understanding of the determinants of Roseobacter diversity and their links to phytoplankton, in particular high DMSP producers (e.g. *Prorocentrum*, *Micromonas* and *Phaeocystis*) and low DMSP producers (e.g. *Thalassiosira*, *Skeletonema* and *Bathycoccus*). Here we employed a substantial dataset of 16S rRNA and 18S rRNA gene sequences from the Australian Microbiome Initiative, collected across a unique continental-scale network of oceanographic time-series sites, to assess spatial and temporal patterns in the MRG and DMSP-producer communities. Our goal was to develop a more comprehensive environmental perspective on the ecology of a fundamentally important group of copiotrophic marine bacteria, their links to phytoplankton, and the marine sulfur cycle.

## Methods

### Sampling locations

To characterise patterns in the MRG relative abundance, diversity and associations with DMSP-producing phytoplankton, samples were collected from seven long-term oceanographic time-series sites situated in different regions of the Australian continental shelf (Supplementary Table S1). These National Reference Stations (NRS) span 30 degrees of latitude, with ship-board sampling conducted on a monthly or quarterly basis as part of the Australian Integrated Marine Observing System (IMOS). The Darwin NRS (12° 24.00 S, 130° 46.08 E) is located 9 km offshore from Darwin Harbour in the tropical north of Australia and experiences a monsoonal tropical climate. The Yongala NRS (19° 18.51 S, 147° 37.10 E) is located 22 km offshore and is located in the Great Barrier Reef (GBR) lagoon. This tropical site is also subject to monsoonal climate involving heavy rainfall events between December to April. The North Stradbroke Island NRS (27° 20.50 S, 153° 33.73 E) is located 12 km north-east of North Stradbroke Island (NSI), Queensland, and is influenced by the warm oligotrophic East Australian Current (EAC), with stratification of the water column and increased salinity between September to April [41]. The Port Hacking NRS (34° 05.00 S, 151° 15.00 E) is a long running time-series (since 1953) situated 7 km offshore, near the city of Sydney (population 4.3 million). This subtropical site is at times a transition zone between the cooler waters of the Tasman Sea and warm water intrusions of the EAC. The site can experience occasional upwellings via eddy and wind-driven currents that are linked with enhanced nutrient availability and phytoplankton biomass [42, 43]. The Maria Island NRS (42° 35.80 S, 148° 14.00 E) is located 9 km east of Maria Island, Tasmania. This temperate station is the southern-most NRS location and sits at the southern-most extent of the EAC, with evidence that longer EAC incursions are leading to rapid ocean warming in the region [41]. The water column at the Maria Island NRS is well-mixed year-round and is reported to have greater seasonal and inter-annual variation of microbial populations relative to subtropical sites on the east coast of Australia [44]. The Kangaroo Island NRS (35° 49.93 S, 136° 26.84 E) is located 10 km west of Kangaroo Island, South Australia. This subtropical site experiences periodic upwelling episodes in the austral winter [41]. The Rottnest Island NRS (32° 00.00 S, 115° 25.00 E) is located 4.5 km from Rottnest Island, which is 22 km off the western Australian mainland in the Indian Ocean. This site is heavily influenced by the Leeuwin Current, which transports warm water southward and as a result, sea-surface temperatures are up to 5 °C warmer at the NRS site compared to systems at similar latitudes (32° S).

### Characterisation of physicochemical conditions

Water samples and accompanying physicochemical data were collected on a monthly basis at the Yongala, Port Hacking, North Stradbroke Island, Maria Island and Rottnest Island NRS, while quarterly sampling was conducted at Darwin and Kangaroo Island between February 2012 and August 2017. Physicochemical parameters including sea-surface temperature (°C) and salinity (PSU), chlorophyll *a*, dissolved oxygen (μmol L^−1^), and inorganic nutrients, nitrate/nitrite (μmol L^−1^), orthophosphate (μmol L^−1^), ammonia (μmol L^−1^), and silicate (μmol L^−1^) were measured in accordance with the IMOS NRS sampling protocol [45, 46] and were retrieved from the IMOS curated Australian Ocean Data Network Portal (https://portal.aodn.org.au/).

### Sample collection and microbial DNA preparation

A detailed description of the marine microbial sample collection protocols within the Australian Microbiome Initiative is provided in Brown et al. [41]. Briefly, 2 L of seawater was collected from pre-determined depths (Supplementary Table S1) using Niskin bottles and filtered onto 0.2 μm pore Sterivex™ GP filter (Millipore, MA, USA). DNA was subsequently extracted from filters using a modified PowerWater^®^ Sterivex™ DNA Isolation Kit (MOBIO laboratories, Carlsbad, CA, now Qiagen) [47].

### Amplicon sequencing and bioinformatic analysis

Bacterial and eukaryotic assemblages were characterised using 16S rRNA and 18S rRNA gene sequencing, respectively. 16S and 18S rRNA amplicon PCR sequencing was performed using the Illumina MiSeq platform at the Ramaciotti Centre for Genomics at the University of New South Wales. The Australian Microbiome project, that commenced in 2011 utilises the V1–V3 hypervariable region of the prokaryotic 16S rRNA gene and differs from other more recent large-scale molecular projects (TARA Oceans Expedition, Malaspina Expedition and BIOGEOTRACES project) that use the V4–V5 region [48–51]. Direct comparisons between these primers are scarce, however, it has been reported that V1–V3 provided considerably higher estimates of SAR11 (an Alphaproteobacteria) abundance compared with estimates provided by V4–V5, although did not report any inconsistencies between Roseobacter diversity or abundance [52]. V1–V3 was amplified using the 27F (AGAGTTTGATCMTGGCTCAG) [53] and 519R (GWATTACCGCGGCKGCTG) primer pairing [54] under the following thermocycling conditions: 95 °C for 10 min; 35 cycles of 94 °C for 30 s, 55 °C for 10 s and 72 °C; followed by a final extension at 72 °C for 10 min. The V4 region of the 18S rRNA gene was amplified using the TAReuk454FWD1 (CCAGCASCYGCGGTAATTCC) and a modified TAReuk-Rev3 (ACTTTCGTTCTTGATYRATGA) primer [55], designed to be less discriminant against Haptophytes than the original TAReuk-Rev3 primer [56]. 18S rRNA was amplified under the following conditions: 98 °C for 30 s; 10 cycles of 98 °C for 10 s, 44 °C for 30 s and 72 °C for 15 s; 20 cycles of 98 °C for 10 s, 62 °C for 30 s and 72 °C followed by a final extension at 72 °C for 7 min. At the time of analysis, the Australian Microbiome Initiative (AMI) marine microbes database consisted of 1307 16S samples (Accession numbers are available in Supplementary Table S2) and 749 18S samples (Accession numbers are available in Supplementary Table S3) that were analysed using the methods detailed below. All sequences are now publicly available at the AMI data portal https://data.bioplatforms.com/organisation/about/australian-microbiome.

In order to derive the highest possible phylogenetic resolution, an unfiltered database of unique sequences was prepared. Briefly, the database was prepared by merging Illumina R1 and R2 reads using FLASH [57, 58]. Sequences with <3 reads per sequencing run were removed in addition to the removal of all sequences containing N’s. Sequences displaying an N at the terminal base were trimmed and retained within the dataset. All unique sequences were identified and mapped in each sample using -fastx_uniques from USEARCH v.10.0.240 [59, 60] to generate a sample by read abundance table. A total of 59,862 unique 16S rRNA sequences and 67,074 unique 18 S rRNA sequences were generated. Unique bacterial sequences were taxonomically classified using the SILVA v132 database with a 50% Bayesian probability cut-off [61, 62] and phytoplankton sequences were classified using the Protist Ribosomal Reference Database (PR2 version 12) [63]. The workflow to process unique sequences from the AMI database is downloadable from https://github.com/AusMicrobiome/amplicon/raw/1.0.0/docs/amplicon_analysis_workflow.docx and a comprehensive rationale for using unfiltered sequences can be found in Supplementary Methods.

To characterise MRG sequences, a library of 89 de-replicated representative 16S rRNA gene sequences derived from MRG genomes were compiled from www.roseobase.org. Previous genomic analyses of the MRG define the group as a paraphyletic subgroup of Rhodobacteraceae [64] and strains within the Roseobacter group have been identified to share 89% identity of 16S rRNA region [1, 65]. Therefore, all sequences annotated as Rhodobacterales were retrieved from our database. Identification of MRGs in our database were then achieved by clustering representative 16S sequences from Roseobase at 89% similarity with all Rhodobacterales sequences (434 bp, V1–V3) using the VSEARCH clustering tool with default parameters [66]. Across the entire dataset, 2222 16S amplicon sequence variants (ASVs) were clustered with known Roseobacters, identifying them as putative members of the MRG. Phylogenetic trees with putative MRG sequences, an outgroup sequence annotated as SAR11 clade 1a and known Roseobacters were assembled to visualise the MRG phylogeny (Supplementary Fig. S1). Phylogenetic trees were constructed using MEGA bioinformatic software (version 7) [67]. First MRG sequence alignments were generated using MUSCLE with default parameters [68] and the tree topology and confidence estimated using the Maximum-likelihood method (500 bootstraps, Tamura-Nei substitution model) [69, 70].

### Identifying DMSP-producing phytoplankton in 18S rRNA sequences

Photosynthetic protists defined as 18S rRNA ASVs assigned to PR2 taxa: Chlorophyta, Dinophyta, Cryptophyta, Haptophyta, Ochrophyta, Cercozoa, Syndiniales and Sarcomonadea were extracted from the dataset, resulting in a subset of 10,875 18S ASVs. A curated bioinformatic pipeline was used to classify 18S ASVs as potential DMSP producers by incorporating previous measurements of cellular DMSP production in monocultures [58] to putatively assign the ability to produce DMSP to 18S ASVs based on phylogenetic inference [71]. First, full length 18S sequences of strains found to contain one (or more) DMSP synthesis gene (DSYB and TPMT1/TPMT2) (*n* = 164) or no DMSP synthesis gene (*n* = 180) were collected from the Marine Eukaryotic Transcriptome Sequencing Project (MMETSP) (Supplementary Table S4) [72] The 18S sequences were aligned with the eukaryotic small subunit ribosomal RNA Rfam (RF01960) using Infernal (v 1.1) [73] in order to build a reference phylogeny with RAxML (v 8.0) [74] using the GTRGAMMA model. A second alignment of the 10,875 unique 18S ASVs was created with Infernal and then pplacer [75] was used to place ASVs onto the reference phylogeny. 18S ASVs that had significant sequence similarity (posterior probability of 90%, likelihood < −4000) with one of the identified MMETSP strains with or without DMSP biosynthesis gene carriers were assumed to be DMSP producers or non-DMSP producers, respectively. A total of 3359 18S sequences were identified as likely DMSP-producing phytoplankton and 1053 18S sequences identified as likely non-DMSP producers (NoDP) (Supplementary Table S5). The DMSP-producing ASVs were further categorised as low DMSP producers (LoDP) or high DMSP producers (HiDP) based on SILVA taxonomic assignment (at Genus level) matching isolates with previously measured intracellular DMSP concentrations of less than, or greater than 50 mM DMSP, respectively [76]. Of the putatively identified DMSP-producing ASVs, 143 were related to 18S sequences of known HiDPs, and 74 were related to 18S sequences of known LoDPs. The pipeline identified 3142 ASVs as DMSP producers that did not belong to a genus of phytoplankton with previously characterised intracellular DMSP concentrations (herein referred to as UnDP). The remaining 18S ASVs (6,460) that were not classified from the pipeline were omitted from subsequent analyses.

### Statistical analyses

To identify differences in the composition of the MRG across sites we performed multivariate Analysis of similarities (ANOSIM) with Bonferroni corrected post-hoc pairwise tests on the 50 most abundant MRG ASVs. To understand relationships between MRG ASVs and environmental parameters (salinity, temperature, ammonium, nitrate/nitrite, oxygen, phosphate, chlorophyll *a* and silicate) responsible for dissimilarity between sites, SIMPER analysis and Canonical Correspondence Analysis were performed on the 50 most abundant MRG ASV normalised abundances. To investigate seasonal and intra-annual variability of the community structure of the top 50 MRG and DMSP-producing phytoplankton the temporal distance between samples (time lag) was compared using Bray–Curtis (B–C) similarities and a Mantel correlogram of all sites by grouping month and year. Seasonal shifts in communities were statistically verified by pooling B–C similarities in 6-month sampling intervals (Austral Autumn-Winter; Mar, Apr, May, Jun, Jul, and Aug, and Austral Spring-Summer; Sep, Oct, Nov, Dec, Jan and Feb) and comparing the average similarity between 6 monthly intervals (e.g. Spring-Summer vs. Autumn-Winter) and twelve-monthly intervals (e.g. Spring-Summer vs. Spring-Summer) using a Kruskal–Wallis test (*p* < 0.05). To test for co-occurrence patterns between the 50 most abundant MRG and DMSP-producing phytoplankton in samples from all seven NRS stations over the years 2015–2017 (*n* = 749 samples), the maximal information-based non-parametric exploration (MINE) pipeline was used. MINE is a tool used to detect novel associations in large datasets and provides a suite of non-parametric exploration statistics that can be used to identify and characterise relationships in data [63]. As a point of reference, the 50 most abundant non-MRG bacterial ASVs were also included in the analysis. To minimise the chance of spurious correlations, only MINE results with a total information coefficient (TIC_*e*_) value greater than zero (indicating presence of relationship) and a maximal information coefficient (MIC_*e*_) value ≥0.178 (indicating a significant relationship, corresponding with a corrected *p* value < 0.001 for *n* = 749) were included [77]. All significant positive correlations (Spearman’s Rho > 0) were used to calculate the average correlation between MRGs/non-MRGs and HiDP, LoDP, and NoDP. Significant differences between the strength of positive correlations (Spearman’s Rho) between the top 50 MRG ASVs and top 50 non-MRG ASVs to HiDPs, LoDPs, and NoDPs were tested using a One-way ANOVA followed by Tukey-Kramer pairwise comparisons with Bonferroni corrections (*q* < 0.05). All non-homoscedastic (Levene’s test, *p* < 0.05) univariate statistical tests used Kruskal–Wallis test with Bonferroni corrected post-hoc comparisons (*q* < 0.05). An additional dataset (*n* = 385) limited by the number of samples that contained all of the desired contextual data (i.e. salinity, temperature, ammonium, nitrate/nitrite, oxygen, phosphate, chlorophyll *a*, and silicate) was included in MINE analysis to identify any significant correlations between MRGs and environmental variables. Strong correlations (adjusted *p* value < 0.001, Spearman’s Rho > 0.6) between MRGs and DMSP producers and MRGs and environmental variable were visualised using an Edge-weighted Force directed function in Cytoscape v3.71. All univariate tests were performed using SPSS version 17.0 (SPSS Statistics, Inc., Chicago, IL, USA) and multivariate tests were performed using PAST version 4.0 [78].

## Results and discussion

### Spatial and temporal patterns in MRG

**Fig. 1:**
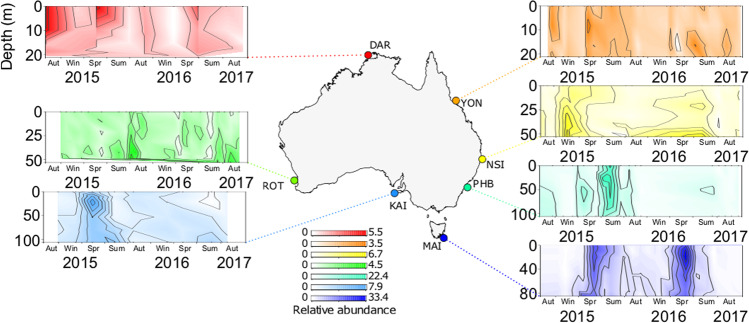
Total relative abundance (%) of the marine Roseobacter group  (2222 unique 16S rRNA sequences) at the seven NRS between Jan′ 15 and Aug′ 17 at Darwin Harbour (DAR, Red), Yongala (YON, Orange), North Stradbroke Island (NSI, Yellow), Port Hacking Basin (PHB, Aqua), Maria Island (MAI, Dark Blue), Kangaroo Island (KAI, Cornflower Blue) and Rottnest Island (ROT, Green).

Our analysis of the overall relative abundance of the marine Roseobacter group (MRG) revealed continental-scale spatial patterns. The relative abundance of total MRG amplicon sequence variants (ASVs) was significantly greater at the subtropical and temperate sites of Port Hacking and Maria Island (Kruskal–Wallis, *q* < 0.05) compared to the other sites around Australia (Fig. 1). On average, MRG ASVs comprised 6.2 ± 0.34% and 5.7 ± 0.33% of the total bacterial community at Maria Island and Port Hacking respectively, whereas the lowest average relative abundance of MRG ASVs (1.8 ± 0.05%) was observed at the tropical site Yongala (Fig. 1). Overall, our results corroborate previous research showing that the MRG is widely distributed in marine environments and typically constitute 2–8% of surface water bacteria [2, 3]. The peak relative abundance of MRGs measured in samples at Maria Island (33.4% of total bacteria) and Port Hacking (22.4% of total bacteria) occurred above the thermocline (between 0 and 25 m depth) (Fig. 1). Patterns of increased MRG relative abundance in two of the sites on the south-eastern Australian coastal shelf (Port Hacking and Maria Island) might be explained by an overlap of environmental niches shared by dominant groups of the MRG, such as the CHAB-I-5 cluster [14, 40] and the Roseobacter clade affiliated cluster [13].

Temporal patterns in total MRG ASV relative abundance were also evident with significant differences found between months at all sites (Kruskal–Wallis, *q* < 0.05). Strong increases in total MRG relative abundance were measured in November to December at Port Hacking and between September to January at Maria Island, peaking in November 2015 and November 2016, respectively (Fig. 1). Notably, temporal increases in MRG relative abundance at Port Hacking and Maria Island are within the same range (as much as 20–40% of all 16S rRNA relative abundance) as MRG populations during spring-time phytoplankton blooms in other coastal locations [79, 80]. Exploration of the relationship between MRG relative abundance and phytoplankton biomass (chlorophyll *a* (chl *a*)) revealed significant positive correlations between these variables at Yongala, Rottnest Island, Kangaroo Island, Port Hacking and Maria Island (Supplementary Fig. S2). Furthermore, peaks of chl *a* at Port Hacking (16.9 mg chl *a* L^−1^) and Maria Island (1.6 mg chl *a* L^−1^) coincided with the MRG abundance comprising over 20% of the total bacterial abundance (Supplementary Fig. S2).

**Fig. 2:**
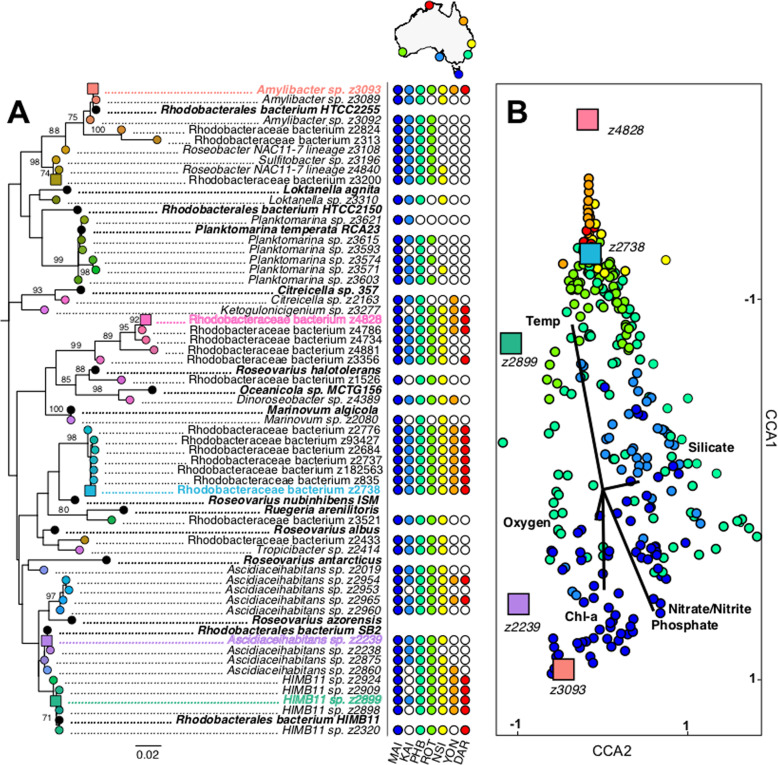
Spatial and phylogenetic distribution of dominant marine Roseobacter group sequences (MRGs) across time-series locations. **A** Maximum-Likelihood phylogeny of 50 most abundant MRGs with known Roseobacter representative sequences (in bold). Presence (filled dot) or absence (unfilled dot) indicated at each time-series location. Coloured text corresponds with MRCs presented in panel **B**. Bootstrap values >70 are shown. **B** Canonical Correspondence Analysis (CCA) of top 50 MRG communities at DAR (Red), YON (Orange), NSI (Yellow), PHB (Aqua), MAI (Blue), KAI (Cornflower Blue) and ROT (Green). Vectors represent environmental parameters. The coloured boxes in CCA plot represent top 5 Roseobacters contributing to dissimilarity amongst MRG communities. Sequences of abundant MRG ASVs and known representatives in Supplementary Table S5.

To further examine spatial and temporal patterns in the MRG we focused on a subset of the 50 relatively most abundant MRG ASVs across the entire dataset (Fig. 2A, Supplementary Table S6). These ASVs accounted for more than 75% of total MRG assigned 16S rRNA reads (2260 ASVs). All targeted ASVs were present at the temperate site, Maria Island, while only 19 of these ASVs occurred in the tropical site at Darwin (Fig. 2A). Significant dissimilarities in the composition of the targeted MRG communities were found between study sites (Fig. 2B, ANOSIM, *R* = 0.3758, *p* = 0.0001). The stations at Yongala, Kangaroo Island, and Maria Island each hosted unique MRG communities compared to all other sites (*q* < 0.05). The four remaining MRG assemblages at Darwin, Rottnest Island, North Stradbroke Island and Port Hacking did not differ from one another with exception of significantly different MRG communities at Port Hacking relative to Rottnest Island (*q* < 0.05). The latter pattern highlights differences between the Pacific Ocean and Indian Ocean bacterial communities, despite the sampling sites differing by only 2 degrees of latitude and both being characterised by subtropical conditions.

**Fig. 3:**
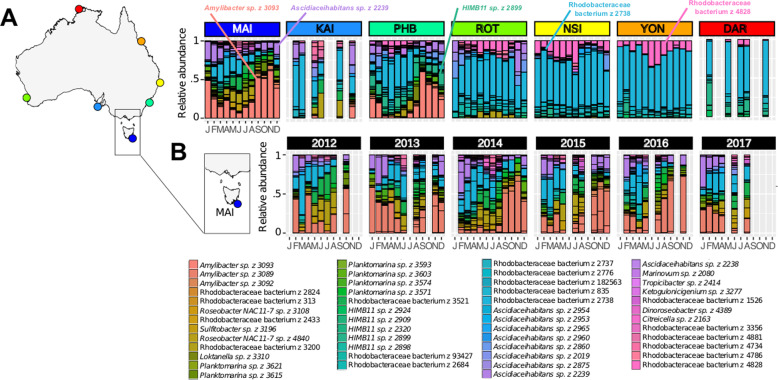
Temporal distribution of dominant marine Roseobacter group (MRG) ASVs. **A** Average monthly relative abundance of 50 most abundant MRG sequences in surface waters (<5 m depth) in the Australian Microbiome time-series between 2015 and 2017*. **B** Monthly relative abundance at Maria Island, Tasmania between Feb 2012 and Aug 2017. *2012–2017 for MAI, PHB and NSI.

Among environmental parameters, temperature, chlorophyll *a*, nitrate/nitrite and orthophosphate were variables correlated to MRG community dissimilarity (Fig. 2B). Temperature was most strongly correlated with MRG communities at Darwin, Yongala, North Stradbroke Island and Rottnest Island (Fig. 2B). Meanwhile, nutrients and chl *a* were correlated with MRG communities at Port Hacking, Kangaroo Island and Maria Island (Fig. 2B). Five abundant MRG ASVs collectively contributed to half of the average dissimilarity between sites (Fig. 2A, Supplementary Table S7). Biogeographical differences existed between the major contributors of dissimilarity (Fig. 2B). The bacterium (z2738) was the most abundant MRG across the entire dataset, representing an average of 43 ± 2.5% of the dominant MRG community (Fig. 3A). Rhodobacteraceae bacterium (z2738) was the dominant member of the MRG at subtropical (Rottnest Island and North Stradbroke Island) and tropical sites (Yongala and Darwin) that were correlated with temperature (Fig. 2B), along with Rhodobacteraceae bacterium (z4828) and *HIMB11* (z2899) (Fig. 3A). Dominant members of the MRG at Port Hacking, Kangaroo Island and Maria Island were *Amylibacter* sp. (z3093) and *Ascidiaceihabitans* sp. (z2239) (Fig. 3A). *Amylibacter* sp. (z3093) was the most abundant ASV at Port Hacking and Maria Island, representing 20 ± 5.4% and 31 ± 5.5% of the dominant MRG community at the sites correlated to chl *a* and nutrients (phosphate and nitrate/nitrite) (Fig. 2B).

**Fig. 4:**
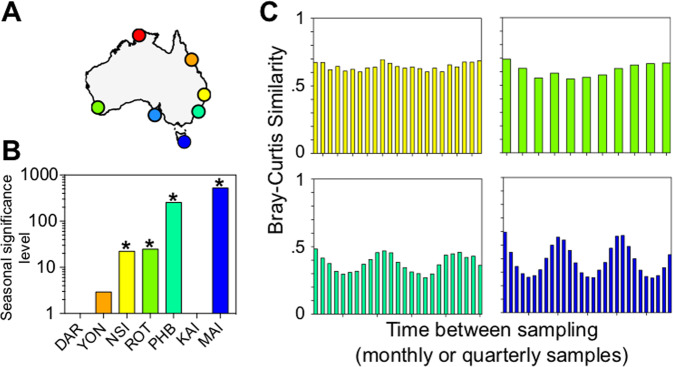
Seasonal shifts in top 50 marine Roseobacter group (MRG) community. **A** Australian National Reference Stations; Darwin (DAR, red), Yongala (YON, orange), North Stradbroke Island (NSI, yellow), Rottnest Island (ROT, green), Port Hacking (PHB, aqua), Kangaroo Island (KAI, cornflower blue) and Maria Island (MAI, dark blue). **B** Independent samples Kruskal–Wallis Test Statistic testing the null hypothesis of H_0_ = No difference exists in average Bray–Curtis Similarities between 6 monthly sampling and yearly sampling comparisons (Supplementary Table S8). Bars with asterisks (*) indicates that significant seasonal shifts are present. **C** Bray–Curtis similarities of top 50 MRG communities in all pairs of samples (*y*-axis) separated by different intervals of time (*x*-axis) in sites with significant seasonality.

In addition to biogeographic differences in the MRG community, temporal variability in the top 50 most abundant MRG ASVs was apparent at several of the sites, with levels of seasonality varying between sites (Fig. 3A). No seasonal variability in MRG community was evident in the tropical time-series at Darwin and Yongala (Fig. 4B, Supplementary Table S8). At these sites, similarities between sample pairs (time lags) ranged from 99.6 to 99.9% at Darwin and 75 to 80% at Yongala (Supplementary Fig. S3). On the other hand, seasonal changes in the MRG community, indicated by significantly lower similarity (*q* < 0.05) between communities at 6-month intervals relative to twelve-month intervals, occurred at the subtropical sites North Stradbroke Island, Rottnest Island and Port Hacking, and the temperate site at Maria Island (Fig. 4B, Supplementary Table S8). The greatest levels of seasonality were observed at Maria Island and Port Hacking, where intra-annual variability of MRG communities ranged from 25 to 60% and 27 to 48% similarity between sampling events, respectively (Fig. 4C). Seasonal differences at Port Hacking were punctuated by peaks in the relative abundance of Rhodobacteraceae bacterium (z2738), whereby this ASV represented 46.2% of the MRG community in autumn-winter, and peaks in the relative abundance of *Amylibacter* sp. (z3093), which made up almost 60% of the MRG community during spring-summer months (Fig. 3A). Similarly, at Maria Island, differences in the MRG community were also driven by *Amylibacter* sp. (z3093), which demonstrated significant increases in relative abundance (up to 62% of the community) during the Austral spring and summer months (Fig. 3B, ANOVA, *F*_3_ = 12.782, *p* < 0.05) and by *Ascidiaceihabitans* sp. (z2239), which displayed significant increases (up to 23% of the community) in relative abundance during the Austral summer (Fig. 3B, Kruskal–Wallis, *q* < 0.05). The strong seasonality in the dominant MRG community and significant positive correlations between total MRG relative abundance and chl *a* (Spearman’s Rho = 0.46, *p* < 0.01, *n* = 324, Supplementary Fig. S2) at Maria Island provides evidence that phytoplankton-derived DOM governs the dynamics of the MRG community in an environment known for predictable annual spring-summer phytoplankton blooms [45, 81].

### Spatial and temporal patterns in DMSP-producing phytoplankton

**Fig. 5:**
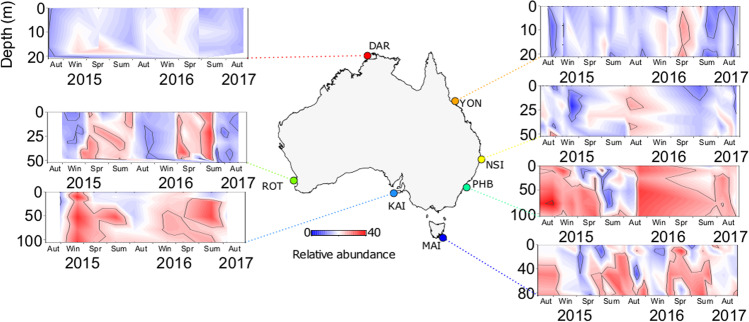
Total relative abundance (%) of DMSP-producing phytoplankton (3359 unique 18S rRNA sequences) in the Australian Microbiome Initiative Time-Series between Jan′ 15 and Aug′ 17 at locations, Darwin Harbour (DAR, Red), Yongala (YON, Orange), North Stradbroke Island (NSI, Yellow), Port Hacking Basin (PHB, Aqua), Maria Island (MAI, Dark Blue), Kangaroo Island (KAI, Cornflower Blue) and Rottnest Island (ROT, Green).

**Fig. 6:**
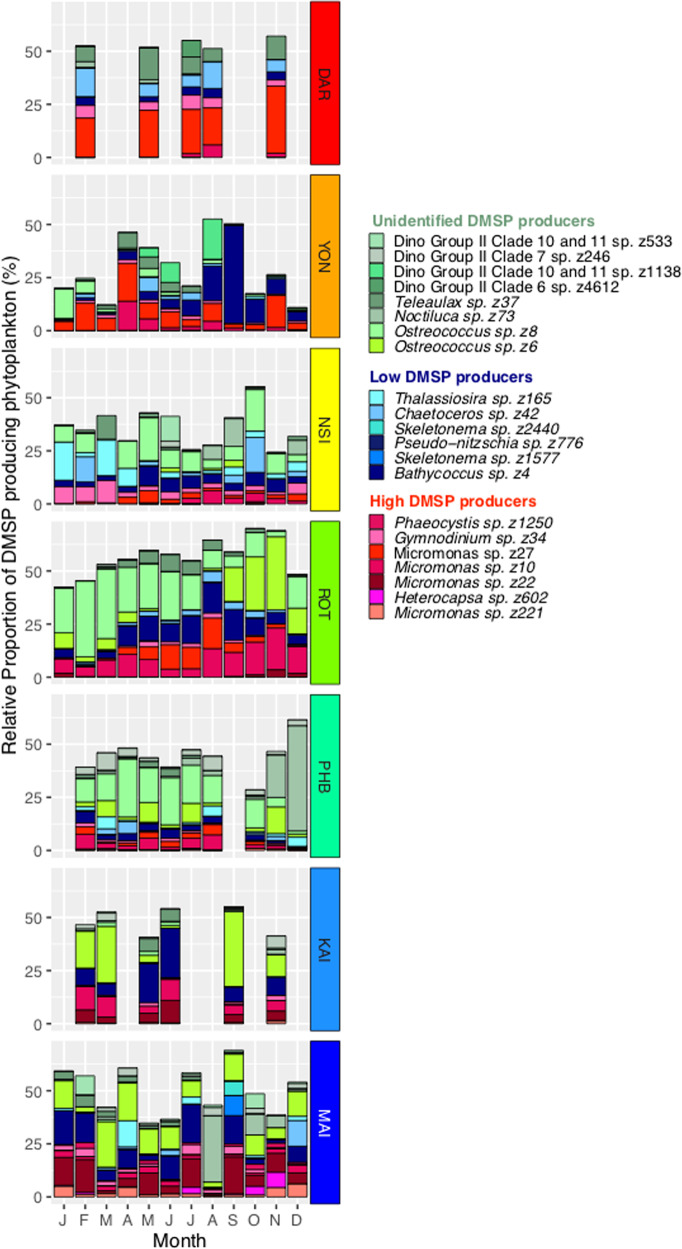
Average monthly relative abundance of 20 most abundant DMSP-producing phytoplankton (all depths) at Australian National Reference Stations; Darwin (DAR, red), Yongala (YON, orange), North Stradbroke Island (NSI, yellow), Rottnest Island (ROT, green), Port Hacking (PHB, aqua), Kangaroo Island (KAI, cornflower blue) and Maria Island (MAI, dark blue) between 2015 and 2017.

To identify the occurrence of biogeographically and temporally conserved associations between MRGs and DMSP-producing phytoplankton, we characterised temporal and spatial patterns in DMSP producers and related these to MRG community dynamics. The spatial distribution of 3,350 ASVs identified as DMSP producers displayed similar continental-scale patterns as the MRG community, with greater total relative abundances (of the 18S phototrophic community) measured in subtropical and temperate waters than in tropical locations (Fig. 5). The greatest relative abundance of DMSP producers was seen at Maria Island, where DMSP-producing phytoplankton comprised an average of 15.2 ± 0.6% (at times representing up to 42%) of the 18S rRNA community (Fig. 5). The dominant DMSP-producer at tropical sites (Darwin and Yongala) was the high DMSP-producer (HiDP), *Micromonas* sp. (z27), which represented an average 2.6 ± 1.08% and 1.8 ± 2.32% of 18S rRNA sequences, respectively. At subtropical sites (North Stradbroke Island, Port Hacking and Rottnest Island), an increased relative abundance of the potential DMSP-producer *Ostreococcus* sp. (z8) (Unidentified DMSP-producer, UnDP, indicated by our pipeline, although no culture studies have confirmed this) was observed (Fig. 6, Kruskal–Wallis, *q* < 0.05). Meanwhile, significant increases in the relative abundance of the LoDP *Bathycoccus* sp. (z4) and the UnDP *Ostreococcus* sp. (z6) were found in more southerly sites (Kangaroo Island and Maria Island) compared to northern sites (Rottnest Island, Port Hacking, North Stradbroke Island, Yongala and Darwin) (Fig. 6, Kruskal–Wallis, *q* < 0.05). Dominant ASVs belonging to the HiDP genus *Micromonas* were also different across subtropical sites and temperate sites (Fig. 6), The relative abundance of *Micromonas* sp. (z10) was greatest at Rottnest Island (1.8 ± 1.69%, Kruskal–Wallis, *q* < 0.05), whereas *Micromonas* sp. (z22) (2.1 ± 1.75%, Kruskal–Wallis, *q* < 0.05) and *Micromonas* sp. (z221) (0.6 ± 0.89%, Fig. 6, Kruskal–Wallis, *q* < 0.05) were most abundant at Maria Island.

Temporal patterns in total DMSP-producer abundances were also clear at other locations, with highest levels observed at Rottnest Island and Maria Island during the spring-summer periods of 2015–2016 (Fig. 5). Annual peaks in relative abundance of identified DMSP producers at Rottnest Island were associated with a dominance of picoeukaryote ASVs; *Micromonas* sp. (z10) (7.2%), *Bathycoccus* sp. (z4) (2.8%) and *Micromonas* sp. (z27) (2.3%) (Fig. 6) in 2015 (August, 46 m depth), and *Micromonas* sp. (z10) (5.2%), *Bathycoccus* sp. (z4) (1.8%) and *Micromonas* sp. (z22) (1.3%) in 2016 (November, 46 m depth) (Fig. 6). Alternatively, temporal peaks in the relative abundance of the DMSP-producing community at Maria Island were dominated by picoeukaryotes and diatoms in 2015 (December 2015, 20 m depth), including *Chaetoceros* sp. (z42) (5.3%), *Bathycoccus* sp. (z4) (4.2%), *Micromonas* sp. (z221) (4.2%), *Micromonas* sp. (z22) (2.8%) and *Micromonas* sp. (z10) (2.3%) (Fig. 6). Whereas a peak in DMSP-producing ASVs at Maria Island in 2016 (September, 20 m depth) were dominated by a mixed community of picoeukaryote, prymnesiophyte and dinoflagellate organisms, specifically *Heterocapsa* sp. (z602) (2.8%), *Micromonas* sp. (z22) (2.8%) and *Phaeocystis* sp. (z1250) (1%) (Fig. 6).

**Fig. 7:**
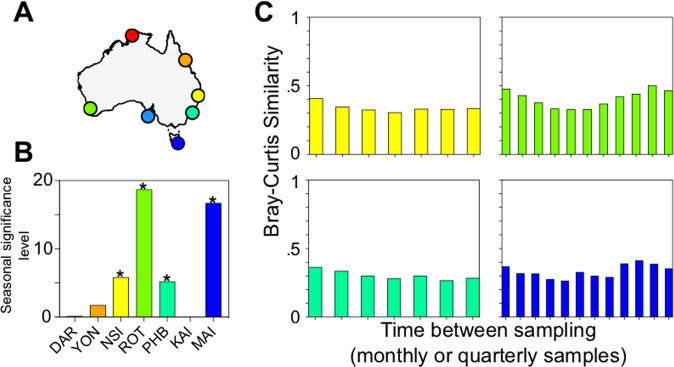
Seasonal shifts in DMSP-producing phytoplankton community. **A** Australian National Reference Stations; Darwin (DAR, red), Yongala (YON, orange), North Stradbroke Island (NSI, yellow), Rottnest Island (ROT, green), Port Hacking (PHB, aqua), Kangaroo Island (KAI, cornflower blue) and Maria Island (MAI, dark blue). **B** Independent samples Kruskal–Wallis Test Statistic testing the null hypothesis of H_0_ = No difference exists in average Bray–Curtis Similarities between 6 monthly sampling and yearly sampling comparisons (Supplementary Table S9). Bars with asterisks (*) indicates significant seasonal shifts are present. **C** Bray–Curtis similarities of top 50 DMSP-producing ASVs in all pairs of samples (*y*-axis) separated by different intervals of time (*x*-axis) in sites with significant seasonality

Consistent trends between MRG and DMSP-producing phytoplankton were not limited to patterns in relative abundance, with greatest levels of seasonality in DMSP-producer diversity observed at the same locations as the largest seasonal shifts in the MRG community (Fig. 7B). Consistent with the patterns in MRG, there was no significant seasonality in DMSP-producer community composition at Darwin, Yongala and Kangaroo Island (Supplementary Fig. S4). Conversely, significant levels of seasonality were observed at Rottnest Island, North Stradbroke Island, Port Hacking and Maria Island (Fig. 7B, Supplementary Table S2).

### Spatiotemporal coupling between Roseobacters and DMSP-producing phytoplankton

Our results indicate the existence of consistent spatial and temporal trends in abundance and levels of seasonality between communities of DMSP-producing phytoplankton and the MRG. Coherent patterns of seasonality between both groups of organisms occurred at several locations (Fig. 7). Moreover, a significant positive correlation (Spearman’s Rho = 0.28, *p* = 0.04, *n* = 749) between the total MRG relative abundance and DMSP-producer relative abundance (Spearman’s Rho = 0.28, *p* = 0.04, *n* = 749) further supports the hypothesis that members of the MRG are influenced by the potential availability of DMSP [15].

**Fig. 8:**
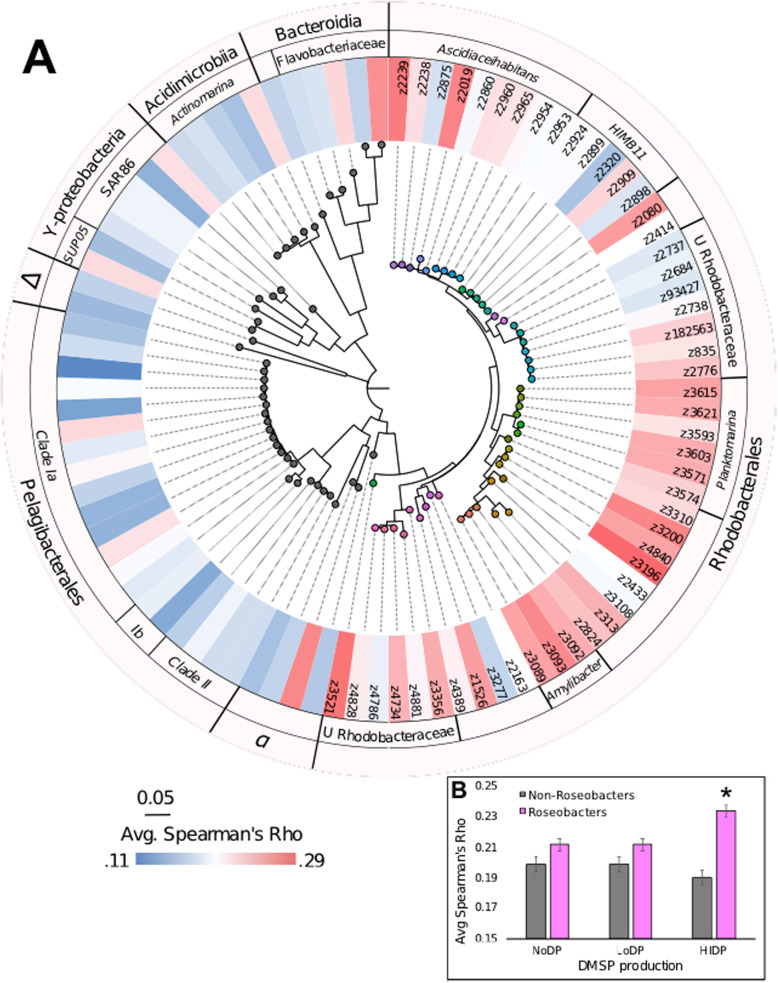
Significant positive correlations between DMSP-producing phytoplankton with heterotrophic bacteria of Roseobacter and non-Roseobacter lineage. **A** Maximum-likelihood tree of top 50 MRGs and 46 most abundant other bacteria in the Australian-Microbiome time-series. Heatmap shows avg. Spearman’s Rho of all significant positive co-occurrences with high DMSP-producing phytoplankton (HiDP). **B** Comparison of avg. Spearman’s Rho between MRGs (pink) and bacteria (grey) with non DMSP-producers (NoDP), low DMSP producers (LoDP) and HiDP. Asterisks denotes significant difference as indicated by Tukey’s HSD (*p* < 0.05). Error bars are mean ± standard deviation.

To more directly examine these potential ecological links, at the level of individual ASVs, we used a network analysis approach. Over 100,000 positive significant interactions were used to generate co-occurrence networks between DMSP-producing ASVs and heterotrophic bacteria. To test the hypothesis that DMSP-producing phytoplankton strongly influence the spatiotemporal dynamics of the MRG, we compared the strength and number of correlations between DMSP producers putatively classified by their potential cellular DMSP concentrations (high DMSP producers (HiDP), low DMSP producers (LoDP) and non-DMSP producers (NoDP) with the 50 most abundant MRGs. Overall, significantly greater average Spearman’s Rank correlations were present between MRGs and HiDPs compared to LoDPs and NoDPs (Fig. 8B). Additionally, no difference in average correlation was found between MRG relative abundance with LoDP or NoDP phytoplankton relative abundance (Fig. 8B). These differences suggest an important role HiDPs play in providing a preferential source of reduced sulfur to MRGs [82, 83]. Moreover, we reveal that LoDP and NoDP phytoplankton demonstrate a weaker impact on MRG abundance, most likely due to the release of lesser or no DMSP as a dissolved organic substrate. We also compared MRG–DMSP-producer correlations to other non-MRG bacterial lineages (e.g. Pelagibacterales, SAR86, Actinobacteria and Flavobacteriales) with DMSP producers. While there were not more correlations between MRG and DMSP producers, the correlations between MRG and HiDPs were significantly stronger than non-MRG lineages to HiDPs, LoDPs and NoDPs (Fig. 8B). This pattern is indicative of specific ecological associations between MRG and high DMSP-producing phytoplankton and is consistent with previous field studies that reported the dominance of Roseobacters in microbial communities associated with blooms of high DMSP-producing phytoplankton species including *Emiliania huxleyi* and *Phaeocystis* sp. and the isolation of Roseobacters from the high DMSP-producer *Alexandrium* sp. in laboratory experiments [10, 35, 84]. Our approach, which has examined a continental-scale dataset comprised of seven molecular time-series, spanning tropical to temperate environments of the Pacific and Indian Ocean, inclusive of two of the four major boundary currents of the Southern hemisphere provides a new and direct line of environmental evidence that Roseobacters are indeed key sulfur cycling lineages associated with prolific DMSP producers.

**Fig. 9:**
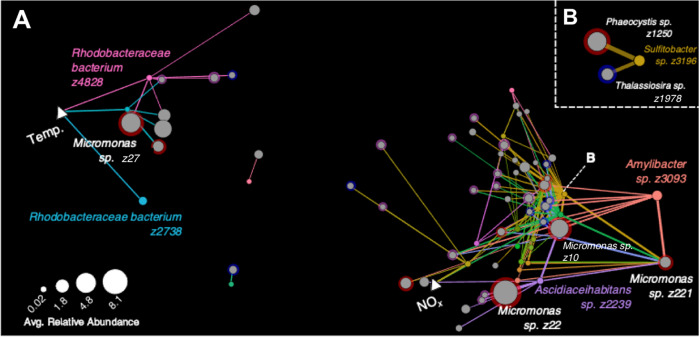
Network Analyses between abundant marine Roseobacter group (MRG) ASVs with DMSP producing phytoplankton ASVs. **A** All Strong positive Roseobacter—DMSP-producer interactions. Coloured nodes show MRGs (as defined in Fig. 3) and grey nodes are eukaryotes. DMSP-producer type is designated by coloured halo to represent high DMSP-producers (HiDP, red), low DMSP-producers (LoDP,  blue), unidentified DMSP-producers (UnDP, pink) and non DMSP-producers (NoDP, no halo). Size of nodes shows average relative abundance of 16S (Roseobacters) and 18S rRNA sequences (Phytoplankton) across all samples (*n* = 749). Nodes are edge-weighted force directed (Biolayout) based on strength of Spearman’s Rho. Thickness of edges represent Spearman’s Rho > 0.6 between an MRG—eukaryote. Environmental parameters shown as triangle nodes (Temp. temperature, NOx nitrate/nitrite). **B** Strongest interactions between *Sulfitobacter* and eukaryotes.

A subset of strong significant positive interactions between the top 50 MRG and DMSP-producing phytoplankton was used to construct a network highlighting ASV-specific ecological interaction in our dataset (Fig. 9). A total of 22 of the 50 MRGs tested with phytoplankton ASVs had at least one significant positive interaction resulting in a Spearman’s Rho of greater than 0.6 (Fig. 9A). Of the phytoplankton ASVs (51 ASVs) with strong correlations to MRGs, ~18% were designated as HiDPs and 16% were LoDPs (Fig. 9A). Overall, 127 strong MRG–phytoplankton interactions (Spearman’s Rho > 0.6) were identified, consisting of 43 correlations to HiDPs and 26 to LoDPs (Fig. 9A, Supplementary Material S1). The strongest correlations across the entire network occurred between the MRG ASV identified as *Sulfitobacter* sp. (z3196) with the HiDP *Phaeocystis* sp. z1250 (Spearman’s Rho = 0.819) and LoDP *Thalassiosira* sp. (z1978) (Spearman’s Rho = 0.792) (Fig. 9B), providing evidence that *Sulfitobacter* sp. (z3196) represents a key associate of DMSP-producing phytoplankton. This *Sulfitobacter* sp. ASV was most prevalent at Kangaroo Island and Maria Island, where on average it represented 2.3% of the MRG community, and 0.2% of the total bacterial community at both sites (Fig. 3A). Notably, this ASV exhibited 5–10-fold increases in relative abundance in parallel to similar increases in relative abundances of *Phaeocystis* sp. z1250, which represented up to 2.4% of the eukaryotic community at Maria Island, and *Thalassiosira* sp. z1978, which represented up to 1.9% of the eukaryotic community at Kangaroo Island. This continental-scale co-co-occurrence between *Sulfitobacter* with DMSP producers, validates repeated identification and isolation of *Sulfitobacter* strains from DMSP-producing phytoplankton cultures [85–87] or blooms [88–90]. Moreover, the result is supported by recent experimental evidence describing the potential mechanism underpinning this co-occurrence, involving metabolic exchanges, including the transfer of DMSP, between *Sulfitobacter* (strain SA11) and the DMSP-producing diatom *Pseudo-nitzschia multiseries* that promoted growth of both partners [16]. The observed co-occurrence patterns across our seven oceanographic time-series are significant as they provide further in situ support for the existence of potentially symbiotic relationships between *Sulfitobacter* sp. and DMSP-producing phytoplankton [16].

Several other dominant MRG ASVs displayed strong correlations to ASVs identified as the marine picoeukaryote *Micromonas*. Specifically, strong correlations involved *Amylibacter* sp. (z3093) and *Micromonas* sp. (z221) (Spearman’s Rho = 0.788), as well as *Ascidiaceihabitans* sp. (z2239) and *Micromonas* sp. (z22) (Spearman’s Rho = 0.748) and Rhodobacteraceae bacterium (z4828) with *Micromonas* sp. (z27) (Spearman’s Rho = 0.64) (Fig. 9A). Notably a laboratory isolate, *Micromonas pusilla* CCMP490 (NCBI accession AY955003.1) is a high DMSP-producing phytoplankton with measured intracellular concentrations as great as 161.9 mmol L^−1^ [76, 91], and is a similar organism (according to 18S rRNA gene sequences) to the ASVs *Micromonas* sp. (z22) (97% identity, 100% query cover), *Micromonas* sp. (z221) (97% identity, 100% query cover) and *Micromonas* sp. (z27) (96% identity, 100% query cover). Little is currently known about DMSP production by picoeukaryotes, as few isolates (*n* = 19 Chlorophyta and *n* = 2 Pelagophyta) have measured cellular DMSP content [58], though the recently reported methyltransferase enzymes required for DMSP synthesis have been detected in in situ picoeukaryote communities [92]. Additionally, previous modelling efforts have found that picoeukaryotes must be important DMSP producers, particularly in oligotrophic conditions, to account for DMSP production when other DMSP producers (e.g. diatoms, haptophytes) are not present/in bloom [76, 93]. Strong correlations between dominant MRG ASVs with abundant *Micromonas* ASVs in our results infer MRGs may display unexpected ecological links to, or even dependencies on, picoeukaryote derived DMSP.

## Conclusion

Here we exploited the power of a continental-scale network of oceanographic time-series to provide a unique in situ assessment of the potential ecological links among marine Roseobacters and DMSP-producing phytoplankton. This approach revealed significant biogeographical and seasonal shifts in the composition of MRG that were often tightly coupled with repeating annual abundance patterns in eukaryotic phytoplankton likely responsible for producing DMSP. While we acknowledge the correlative nature of our results does not deliver irrefutable causal links between the two groups, we contend that large-scale and highly spatially and temporally resolved sampling efforts provide a powerful means of predicting community dynamics and inferring structure, particularly when a priori knowledge of the interaction mechanisms are available [94]. Using this approach, a number of novel and potentially significant ecological associations among often abundant members of the marine microbiome were identified, including new evidence for ecological associations between picoeukaryotes and specific members of the MRG. Our analysis is supported by two decades of laboratory and mesoscale interactions [4, 7, 10, 11, 35, 36, 80, 95–97] and delivers for the first time, ocean-scale field-based confirmation for the important ecological interactions between a key group of copiotrophic marine bacteria and DMSP-producing phytoplankton that are likely to play a pivotal role in carbon and sulfur cycling processes in the surface ocean.

## Supplementary information

Supplementary material

Supp. Fig. S1

Supplementary Tables
