# Supplementary material for: Biogeographical and seasonal dynamics of the marine Roseobacter community and ecological links to DMSP-producing phytoplankton
Source: ISME Commun. 2022 Feb 14;2:16. doi: 10.1038/s43705-022-00099-3 (PMC9723663; doi:10.1038/s43705-022-00099-3)
Supplement: Supplementary file 1 — Supplementary material [file 43705_2022_99_MOESM1_ESM.docx]

**Biogeographical and seasonal dynamics of the marine Roseobacter community and ecological links to DMSP-producing phytoplankton**

James O’Brien*, Erin L. McParland, Anna Bramucci, Nachshon Siboni, Martin Ostrowski, Tim Kahlke, Naomi Levine, Mark V. Brown, Jodie Van De Kamp, Levente Bodrossy, Lauren Messer, Katherina Petrou and Justin R. Seymour*

*Correspondence: [james.obrien@student.uts.edu.au](mailto:james.obrien@student.uts.edu.au) and justin.seymour@uts.edu.au

**Supplementary Methods**

**Rationale for using unfiltered sequences**

The rationale for providing an unfiltered dataset follows the observation that some ecologically significant zOTUs were disappearing from the processed table. The cause was identified as the denoising step (carried out with unoise3 within the AMI amplicon processing pipeline). As an example, within the Order Cyanobacteriia, Genus *Synechococcus*, two ecologically significant clades display are known to display a single base pair change within the 16S SSU rRNA gene V1-V3 region (27F-~520R), i.e clade II (subclades abc) and clade IV. The denoising process resulted in the merger of clade IV into clade II, thereby affecting the accuracy of abundances for each ASV. As a response our analysis moved to unfiltered sequences in an attempt not to lose any ecologically significant sequences.

**Supplementary Figures**

**Fig. S1** Maximum Likelihood Tree of known Marine Roseobacter group representatives with ASVs.

**Fig. S2** Average chlorophyll *a* concentrations (depth-integrated) at each time-series (left). Linear regression between total marine Roseobacter clade (MRC) relative abundance in all bacteria and chlorophyll *a* concentrations. Spearman’s Rho correlation between variables shown for each site (right). Chlorophyll *a* data is derived from CTD measurements and supplemented with high performance liquid chromatography measurements in the absence of CTD data.

**Fig. S3** Mean Bray-Curtis similarities of top 50 MRC communities in all pairs of samples (y-axis) separated by different intervals of time (x-axis).

**Supp. Fig. S4** Mean Bray-Curtis similarities of eukaryotic DMSP-producing communities in all pairs of samples (y-axis) separated by different intervals of time (x-axis).

**Supplementary Tables:**

**Table S1** Site description of time-series locations used. Adapted from Brown et al., 2018.

**Table S2** List of samples used for 16S rRNA analysis (n = 1307).

**Table S3** List of samples used for 18S rRNA analysis (n = 749).

**Table S4** Eukaryotic strains identified in the MMETSP database with and without DMSP biosynthesis genes (*dsyB, tpmt2*) used in pplacer pipeline.

**Table S5** 18S sequences assigned as DMSP-producers by pipeline.

**Table S6** 50 most abundant Roseobacter ASVs and representative sequences derived from Roseobase.org.

**Table S7** Average dissimilarity derived from SIMPER analysis of abundant Roseobacter communities across sites.

**Table S8** Seasonality of top 50 Marine Roseobacter Clade communities in time-series surface waters. Average Bray-Curtis similarities shown from binning all sample pair comparisons of six-monthly and yearly sample intervals. Bold Kruskal-Wallis test statistics and p-values indicate a significant difference between six-monthly and yearly sampling times (p < 0.05). Round parentheses show number of samples at each site. Square parentheses show number of paired sample comparisons used to calculate average similarity.

**Table S9** Seasonality of DMSP-producing phytoplankton communities in time-series surface waters. Average Bray-Curtis similarities shown from binning all sample pair comparisons of six-monthly and yearly sample intervals. Bold Kruskal-Wallis test statistics and p-values indicate a significant difference between six-monthly and yearly sampling times (p < 0.05). Round parentheses show number of samples at each site. Square parentheses show number of paired sample comparisons used to calculate average similarity.
